# Unmet Supportive Care Needs among Breast Cancer Survivors of Community-Based Support Group in Kuching, Sarawak

**DOI:** 10.1155/2016/7297813

**Published:** 2016-04-27

**Authors:** Emmanuel Joseph Fong, Whye Lian Cheah

**Affiliations:** Department of Community Medicine & Public Health, Faculty of Medicine & Health Sciences, Universiti Malaysia Sarawak, 94300 Kota Saramahan, Sarawak, Malaysia

## Abstract

*Background.* Recognizing the needs of cancer survivors is one of the important aspects in healthcare delivery. This study aimed to determine the prevalence of unmet supportive care needs and its associated factors among the breast cancer survivors of community-based support group in Kuching, Sarawak.* Materials and Methods.* This was a cross-sectional study using Supportive Care Needs Survey (SCNS-SF34). All the members of community-based breast cancer support groups in Kuching were invited. A total of 101 respondents were face-to-face interviewed after the consent was obtained. Data was entered and analyzed using SPSS version 20.* Results.* The respondents endorsed* health system and information* domain with the highest mean score (2.48; 95% CI: 2.32–2.64). Top 10 items with “moderate to high” level unmet needs had a prevalence of 14.9% to 34.7% of respondents indicating need. Significantly higher level of unmet needs was associated with survivors who were younger (less than 60 years old), had higher education attainment, were unemployed, had survival duration of up to 5 years, and were undergoing active treatment.* Conclusion*. Systematic delivery of health information which is targeted, culturally sensitive, and linguistically appropriate for addressing younger age, education level, employment status, length of survivorship, and treatment stage should be considered not only at hospital-based setting but also at the community-based support groups.

## 1. Introduction

Breast cancer remains the most common cancer among women worldwide [1]. Similar experience is seen in Malaysia with breast cancer being the most common cancer among women [2]. Advances in detection and treatment modalities have improved the survival rate of women with breast cancer. In Malaysia, the 5-year survival rate among breast cancer patients has seen improvements over the past decades; studies revealed a 5-year observed survival from 58.4% (CI 0.54–0.63) to 75.7% (CI 0.73–0.79) [3]. Because of its high prevalence and relatively good prognosis, the increase in numbers of survivors forms a growing area of clinical interests and research.

A diagnosis of cancer can alter a person's perspective on health and life itself [4]. Female breast cancer survivors are often weighed down by issues of physical lethargy, pain, breast sensitivity, and difficulty to concentrate which were associated with diminished physical functioning and emotional well-being [5]. Their psychological well-being was found to be affected by fear of cancer spread, recurrence, distress from surgery, fear of second cancer, and future tests [6].

Cancer survivors face a wide range of problems during and after their primary treatment which often persists in a chronic, long-term manner [5]. During this period of survivorship, these survivors have multitude of needs which require attention and identification. Recognizing these needs early in the cancer care continuum is important. Therefore, needs assessment should be carried out as it offers three advantages: (i) patient's perceived needs are directly assessed, (ii) the level of need can be identified as well, and (iii) individuals or patient subgroups with higher level of needs can be identified [7]. Furthermore, understanding unmet needs among cancer survivors, across different age groups, gender, regions, cancer types, stages, survival durations, and various other factors, is in line with making healthcare patient-centered.

With the growing number of breast cancer survivors in the milieu of limited healthcare resources, there is a pressing demand to uncover the unmet needs of these survivors so that health systems may prioritize its service delivery in order to be more effective and efficient. Therefore, this study aims to describe the prevalence of unmet needs among breast cancer survivors in Kuching, Sarawak, and to assess relationship between their unmet needs and various associated factors.

## 2. Materials and Methods

This descriptive cross-sectional study was conducted among breast cancer survivors recruited from the community-based nongovernmental organization (NGO) Sarawak Breast Cancer Support Group (SBCSG) in Kuching, Sarawak. Prior permission was obtained from the center in writing and all survivors in the support group were invited to participate in the study. Written informed consent was obtained prior to the interview assisted survey, which was conducted at the center from January 2014 to June 2014. This community-based approach, in which respondents were recruited, ensured that breast cancer survivors, whether they received treatment from public, private, or mixture of health facilities, were captured in this study.

The Supportive Care Framework by Fitch [8] recognizes that about 20% of cancer patients within the healthcare system have unmet needs. Recent studies showed that cancer patients in Taiwan and Hong Kong had prevalence of supportive care needs between 17–33% and 18–46%, respectively [9]. Therefore, in order to estimate the 20% prevalence of unmet supportive care needs (moderate to high needs) with a confidence interval of 95% and a 10% margin of error, an estimate sample size of 62 was required as per the equation of *n* = *z* ^2^ *pq*/*d* ^2^, where *z* = 1.96, *p* = 0.2, *q* = 1 − *p*, and *d* = 0.1 [10]. The eligibility criteria for inclusion in the study were (i) adult Malaysian females aged 18 years and above, (ii) diagnosed with breast cancer (all stages), and (iii) physically and mentally able to participate.

Sociodemographic characteristics of respondents such as age, ethnicity, marital status, cohabitation status, household income, education, and employment status were included in the survey. Medical characteristics such as age at diagnosis, duration of survivorship, cancer stage at time of diagnosis, and current treatment status were collected as well. The 34-item short-form Supportive Care Needs Survey (SCNS-SF34) was adapted and employed in this study to identify the unmet supportive care needs among survivors [11]. Respondents have reported their preference for the SCNS questionnaire over other health-related quality of life questionnaires [12]. Furthermore, the SCNS-SF34 maintained the same constructs which were (i) psychological, (ii) physical and daily living, (iii) sexuality, (iv) health system and information, and (v) patient care and support, while preserving the psychometric properties of the original long-form SCNS [11]. Validation studies have shown that the Cronbach's alpha of the English version was 0.86–0.96 [11].

The 34-item Supportive Care Needs Survey (SCNS-SF34) offered an assessment of needs in cancer patients via five analytically derived domains: (i) psychological (10 items), (ii) physical and daily living (5 items), (iii) sexuality (3 items), (iv) health system and information (11 items), and (v) patient care and support (5 items). For each item, the respondents were required to indicate their level of need for help over the past one month in relation to having cancer. The level of need would be scored on a five-point Likert scale: 1, no need, not applicable; 2, no need, satisfied; 3, low need; 4, moderate need; and 5, high need. For each item, the respondents are dichotomized to as having “moderate to high” level of need if they scored options 4 or 5 or “no to low” level of need if they scored options 1, 2, or 3. The items with highest percentage of respondents reporting “moderate to high” level of need are then ranked and compared. The domain score was obtained by summing up the responses to each of the items within the domain and dividing the sum by the number of items in the domain. A higher mean score would indicate higher perceived need. For the purpose of analysis, the sociodemographic and medical characteristics of respondents were organized and compared with the supportive care needs domain mean scores using independent *t*-test and one-way analysis of variance (ANOVA). The collected data was then entered and analyzed using the IBM Statistical Product and Service Solutions (SPSS) statistics program version 20. Inferential statistics were generated to answer the study objective based on *p* value of less than 0.05 (*p* < 0.05).

This study was conducted with approval from the Medical Ethics Committee, Faculty of Medicine and Health Science, Universiti Malaysia Sarawak (UNIMAS), Malaysia [UNIMAS/TNC(AA)-03.02/06-11 Jld. 3(12)].

## 3. Results

A total of 101 female breast cancer survivors participated in this study. The average age of respondents was 57.9 (SD 9.53) years. Majority of them were married (79.2%) and Chinese (74.3%). More than half of them were Christians (53.5%). Most of the respondents stay with 2 to 4 other persons at home (56.4%) and have an average household income of between RM 3001 to RM 5000 (43.6%). Approximately 80% of the respondents had primary and secondary school level education. Majority of the respondents were unemployed (70.3%) which include survivors who were housewives and retired from employment.

The mean age at first diagnosis of breast cancer among the respondents in this study was 49.7 (SD 8.45) years with 54.5% of the respondents diagnosed at the age 50 years and older. Mean duration of survivorship was 8.2 (SD 5.72) years. Nearly two-thirds (63.4%) of the respondents had a survival duration of more than 5 years. Approximately 80% of the respondents had early stage (Stages I and II) breast cancer at time of diagnosis. Among the respondents, 72.3% were no longer undergoing any active treatment at time of study and were only on annual outpatient department follow-ups. The other information on the sociodemographic and medical characteristics of the respondents is depicted in Table 1.

Domain mean scores are identified by summing up the responses to each of the items within the domain and dividing the sum by the number of items in the domain. A higher score (maximum 5.00, minimum 1.00) would indicate higher level of needs in the domain.  Table 2 revealed that* health systems and information* domain was found to have the highest mean score (2.48; 95% CI, 2.32–2.64), followed by* psychological* domain (2.01; 95% CI, 1.91–2.12) and* patient care and support* domain (1.93; 95% CI, 1.83–2.03). Meanwhile, the* sexuality* domain has the lowest mean score (1.57; 95% CI, 1.44–1.70).

The top 10 items that respondents have indicated a “moderate to high” level of need for help are as depicted in Table 3. Overall, 9 out of the top 10 items of unmet needs were from the health systems and information domain with only one item from the psychological domain. The highest ranked items were* having one member of hospital staff with whom you can talk about all aspects of your condition, treatment, and follow-up* (34.7%),* being given explanations on those tests about which you would like to get explanations* (29.7%), and* having access to professional counseling (e.g., psychologist, social worker, counselor, and specialist nurse) if you, family, or friends need it* (27.7%).

The ten items with lowest “moderate to high” level unmet needs among the respondents are as shown in Table 4. Overall, the listed items had only less than 5% of the respondents indicated “moderate to high” level of need for help. Five out of 10 of these items are from the psychological domain, followed by sexuality domain (2 items), patient care and support domain (2 items), and physical and daily living domain (1 item). Items with the lowest percentage of respondents reporting needs were* feeling down or depressed *(1%),* feelings of sadness *(1%),* changes in sexual feelings *(1%),* changes in your sexual relationships *(1%), and* more choice about which hospital you attend *(1%).

The supportive care needs (SCNs) domain mean scores are compared to the sociodemographic and medical characteristics of the respondents in Table 5. Respondents age below 60 years (*n* = 57, 56.4%) reported significantly higher mean score across* physical and daily living* domain (*p* = 0.009),* psychological* domain (*p* = 0.002),* sexuality* domain (*p* < 0.001), and* patient care and support* domain (*p* = 0.038) compared to survivors aged 60 years and older (*n* = 44, 43.6%). Ethnic Malays and Sarawak indigenous group recorded higher mean score in the* physical and daily living* domain (*p* = 0.017) and* sexuality* domain (*p* = 0.028) compared to the Chinese respondents. Survivors who are married reported higher mean score in the* sexuality* domain (1.69, 95% CI: 1.54–1.84) compared to those who are never married/widowed/divorced/permanently separated (1.11, 95% CI: 0.97–1.25) (*p* < 0.001). Respondents with primary education level reported a significantly lower domain mean score compared to those with postsecondary or tertiary education level across the domains of* physical and daily living* (1.71 versus 2.19, *p* = 0.012),* sexuality* (1.33 versus 1.95, *p* = 0.007),* patient care and support* (1.76 versus 2.21, *p* = 0.014), and* health system and information* (2.37 versus 3.00, *p* = 0.035). Respondents who were unemployed indicated a higher mean score in both the* psychological* domain (2.18 versus 1.94, *p* = 0.042) and the* patient care and support* domain (2.13 versus 1.85, *p* = 0.034) compared to those who were still employed.

Meanwhile, when comparing the SCNs domain mean scores to the medical characteristics and disease stages of the respondents in Table 5, significantly higher mean scores were reported by respondents with survival duration of 5 years or less compared to those who have survived more than 5 years in the* physical and daily living* domain (*p* < 0.001),* psychological* domain (*p* < 0.001),* sexuality* domain (*p* = 0.019), and* patient care and support *domain (*p* = 0.008). Treatment status among respondents showed significant difference in mean scores across all 5 domains with survivors undergoing active treatment indicating a higher mean score compared to those who are not undergoing any current active treatment (*p* value range from <0.001 to 0.019). There were no significant differences in supportive care needs domain mean scores among respondents with various cohabitation statuses, household income level, and age at diagnosis and between respondents who had early stage cancer compared to those with late stage cancer.

## 4. Discussion

The present study is a cross-sectional analysis of the perceived needs among breast cancer survivors in Kuching, Sarawak, Malaysia. The survivors recruited were generally less than 60 years old, were mainly Chinese, were married, received at least secondary level education, and were unemployed (Table 1).

The age at diagnosis (mean age 49.7) and staging (early stage, 80.6%) were closely similar to various studies done in Malaysia [2]. The mean duration of survivorship which was 8.2 years and was higher in this study compared to 6.7 years in another local study [13]; this could be attributed to the fact that there were more later stage cases recruited from the hospital-based medical records in that study.

The current study (Table 2) revealed that survivors indicated a higher mean score (higher scores representing a higher level of unmet needs in the domain, range 1.00–5.00) in the* health system and information *domain (2.48, 95% CI: 2.32–2.64), followed by* psychological *domain (2.01, 95% CI: 1.91–2.12), and* patient care and support* domain (1.93, 95% CI: 1.83–2.03). Meanwhile,* sexuality* domain was ranked lowest (1.57, 95% CI: 1.44–1.70). This finding is consistent with recent research among breast cancer survivors in Singapore [14], Hong Kong [15], and Korea [16] which ranked the* health system and information *domain with the highest mean score. This tendency towards informational needs has been studied and established [17], with recent systematic reviews suggesting that Asian women reported higher informational needs compared to Western women [18]. Furthermore, the current finding could suggest a dissatisfaction among survivors with information received during follow-up care [16].

Based on individual item ranking (Table 3), there is a greater need expressed within the* health system and information *domain compared to the other domains such as* psychological* domain. These findings however were in contrast to multiple Australian-based research outcomes, in which cancer survivors ranked the* psychological *domain as area with highest level unmet needs [11, 19]. Such difference in prevalence has been established in recent systematic review [18] and could be a reflection of underlying cross-continental cultural difference between survivors of Asian compared to Australian origin.

Furthermore, needs among survivors are dynamic and may change over time. Researchers discovered a shift in perceived needs from informational needs to psychological needs among cancer survivors, citing improvements in information delivery over the years, which have led to the shift [20]. Assuming this trend holds true for cancer survivors worldwide, the current trend in this study indicates much needs to be done in addressing the informational needs of our survivors. Nevertheless, a less emphasis on* psychological* domain in this study sample could be influenced by the fact that the study population was from a community-based breast cancer support group, in which their psychological and emotional needs would probably have been addressed. This finding highlights the importance of proper support group among cancer survivors [21], as well as having breast cancer survivors as support members [12].

Younger respondents aged below 60 years old reported higher level of unmet needs across all 5 domains compared to their older counterparts (Table 5), with significant differences seen in the domains of* sexuality*,* psychological, physical and daily living*, and* patient care and support. *This observation is congruent with studies by researchers in Singapore, which demonstrated that the level of need is higher among survivors aged below 60 years [22]. Similarly Australian cancer patients aged 31–60 years endorsed higher levels of unmet needs in* sexuality*,* psychological, patient care and support, *and* health system and information* domains compared to those who are 70 years or older [20]. Comparable conclusions were drawn from a qualitative study which explored the psychosocial needs of breast cancer survivors in which younger women reported a higher level of needs in terms of support, psychological, practical, physical and information needs [23]. Such disparity may reflect the difference in attitudes between younger adults who are more vocal of their unmet needs than older adults who believe that they should have better coping capacity and thus keep things to themselves [20]. Additionally, a diagnosis of cancer in younger adults may impart a sense of perceived loss in all areas of life, such as physical, psychosocial, emotional, sexuality, and the need for support, which give rise to higher magnitude of needs compared to older adults who may well have adjusted to changes in their life as they age. There were, however, no significant differences (*p* = 0.643) in mean scores within the* health system and information* domain which may suggest that, across all age group, the informational need is ever present once a person is diagnosed with cancer.

Significant difference in mean domain scores was found between Malays and Sarawak indigenous groups compared to ethnic Chinese survivors in both the* physical and daily living *(2.18 versus 1.84, *p* = 0.017) and* sexuality* (1.81 versus 1.48, *p* = 0.028) domains. This variation could be influenced by the fact that almost 75% of the respondents in this study were of ethnic Chinese group, and the number of Malays and Sarawak indigenous groups were inadequate to be representative. In the absence of adequate research information, this finding is inconclusive. There may be an element of cultural background and variation in health beliefs which influence the level of needs in these domains. Further exploration into this area is needed to understand these variations. Comparison with other studies involving ethnic Chinese survivors revealed similar lower scores in* sexuality* domain [9, 14] and* physical and daily living *domain [22]. Lower* sexuality* domain scores could be due to underreporting of sexuality needs, as it is regarded as an intimate matter and not for discussion within the Asian Chinese community. Specific cultural norms among the Chinese could also account for lower* physical and daily living* needs, as it is believed that personal problems or outcry for help reflects badly on the family and may bring shame to the family name [24]; therefore, this may lead to a lower report for need in this domain among the Chinese-majority sample of this study.

Higher level of unmet needs in the* sexuality* domain was endorsed by survivors who are married compared to others (*p* < 0.001). This was consistent with a Korean study [25] whereby married patients (*p* = 0.03) were significantly more likely to indicate the need for help in this domain. Furthermore, this finding makes intuitive sense, given that married survivors have spouse in whom they need to confront their sexuality needs more frequently, hence giving rise to needs.

Higher education attainment was associated with greater level of unmet needs among the respondents (Table 5) with mean domain scores being significantly different between respondents with primary level compared to postsecondary or tertiary level education in* psychological*,* sexuality*,* patient care and support*, and* health system and information* domains. This finding is in agreement with other studies which concluded that education level was found to be predictor of higher level of unmet needs among breast cancer survivors [25, 26]. Survivors with higher level of education are more aware and receptive of their underlying conditions, this has led to their need to actively seek ways to improve their current state of health, and the barriers which they face would be reflected in the domain scores.

Current study revealed that survivors who were unemployed reported a higher domain mean score across all 5 domains compared to those who were employed (Table 5) with significant differences reported in the* psychological* and* patient care and support *domains. There may be underlying factors which influence this outcome. A survivor may be unemployed due to various reasons. Among them could be complications from treatment of breast cancer with residual deficits, which would hamper their ability to work and thus be reported as “unemployed.” Such disadvantage may increase their needs in the* psychological* and* patient care and support *domain.

In this study, shorter duration of survivorship was significantly associated with higher domain mean score across all supportive care needs domains except the* health system and information* domain (Table 5). This is in agreement with recent systematic review which suggests shorter time since diagnosis predicts higher level of needs [18]. These findings can be attributed to the fact that newly diagnosed cancer patients had greater physical and emotional needs compared to those who were already receiving posttreatment follow-up care [27]. This universal observation illustrates the need for concerted effort at addressing unmet needs across various domains early in cancer survivorship. Furthermore, independent of survival duration, informational needs among current survivors are persistent throughout the continuum of survivorship care with no differences seen between younger or older survivors. This could be an indication highlighting the gap in information flow between providers and patients. Closing this gap, a systematic approach in information delivery across the period of survivorship with focus on different aspect of needs and its change in relation to survival duration is needed.

Being under active treatment is significantly associated with higher mean scores across all 5 domains in this study (*p* value range <0.001 to 0.019) (Table 5). This finding is consistent with other studies whereby patients under treatment reported high level of unmet needs [28] while those in remission reported fewer unmet needs in the psychological, information, daily living, and patient care domains [20]. Additionally, recent findings among breast cancer survivors showed that those who were not in remission were associated with unmet needs in* health system and information* and* patient care and support* domains suggesting that these groups of survivors are likely to be receiving intermittent treatment and had to deal with symptom management [19]. Greatest difference in mean scores was observed in the* physical and daily living *domain (2.33 versus 1.77, *p* < 0.001). This difference can be attributed to the fact that survivors receiving active treatment were still coping with physical side effects of treatment modalities such as surgery and chemotherapy and hence had much greater need in this domain compared to those who were not on any active treatment any more.

There was no significant difference in mean scores across all 5 domains in relation to cohabitation status of the survivors (*p* = 0.097–0.866) (Table 5). This finding is different from other studies which concluded that generally cancer survivors living alone reported higher level of unmet needs [29], and breast cancer survivors living alone reported significantly more needs in area of patient care domain (*p* = 0.044) [25]. This could be influenced by the fact that the sampled survivors were recruited from a community-based breast cancer support group, and these survivors being part of this group have had some of their needs satisfied. This could lead to a less obvious discrepancy among the samples.

Household income level did not show any significant difference in any of the 5 domains mean scores. This finding differs from a Korean study which concluded that less income predicted higher needs in* physical and daily living* domain (*p* = 0.019) [25]. It was suggested that having less income leads to financial barriers in terms of ability to satisfy daily personal care and physical needs [25]. Majority of these survivors receive their initial management and follow-up care from the public funded government health facilities which are considered to be relatively free, and therefore the income level within the current healthcare system does not make much impact.

Current study revealed that, in relation to cancer stage at time of diagnosis, there was no significant difference in mean scores across all domains (*p* value range 0.627 to 0.964). This however was different from other studies which reported that tumor size was significant in predicting needs in the* psychological* and* health system and information *domains [25]. Nevertheless, this study suggests that, independent of cancer staging at time of diagnosis, there are still unmet needs to be addressed, with highest mean score in the* health system and information* domain, Early stage (2.48, 95% CI: 2.30–2.66) and later stage (2.42, 95% CI: 1.98–2.85). The present finding may highlight a lack of understanding among survivors about the differences of cancer staging which also is indirectly reflected in the high level of unmet needs in the* health system and information* domain. On the other hand, there may be some form of underlying passive coping mechanism in play, whereby survivors choose to keep themselves occupied and not to think too much about their condition [25]; therefore the differences in staging (early or later state) do not weigh in on their unmet needs mean scores.

The present study has several limitations. First, it was conducted among survivors in a community-based support group, whereby participation was based on voluntary basis and representation from late stage (Stages III and IV), that survivors may be less than expected. This could be attributed to the fact that late stage survivors have shorter life span and might not be in the best health to participate regularly in support group activities. Second, the Chinese-majority ethnic representation in this study, despite not being the majority population in the country, concurs with other local studies and characterizes the higher breast cancer incidence rate among the Chinese in Malaysia. Third, the inherent limitation of being a cross-sectional study means that cause-effect relationships could not be assessed, and changes in unmet needs could not be evaluated over time. Despites these limitations, this study has its strength in the fact that samples were drawn from community-dwelling breast cancer survivors which is more likely to represent their needs in the actual lived-in environment compared to samples obtained purely from hospitals.

In conclusion, this study provided valuable insights into the characteristics of breast cancer survivors in Kuching, Sarawak, and their associated unmet supportive care needs with gaps in the informational domain emphasized. Efforts should include the systematic delivery of health information which is targeted, culturally sensitive, and linguistically appropriate, especially to breast cancer survivors who were younger, had higher education attainment, were unemployed, had survivorship of 5 years or less, and were undergoing active treatment. Future research should recruit samples from both community-based support group and survivors receiving treatment. A longitudinal exploration of the supportive care needs relative to survival duration is also recommended.

## Figures and Tables

**Table 1:** Sociodemographic and medical characteristics of respondents (*N* = 101).

Sociodemographic and medical characteristics	*n*	%	Mean (SD)
Age (years)			57.9 (9.53)
Below 60	57	56.4	
60 and above	44	43.6	
Ethnicity			
Chinese	75	74.3	
Malays and Sarawak indigenous group	26	25.7	
Religion			
Christianity	54	53.5	
Buddhism	25	24.8	
Islam	20	19.8	
No religion	2	2.0	
Marital status			
Married	80	79.2	
Never married/widowed/divorced/permanently separated	21	20.8	
Cohabitation status			
Lives alone or with 1 other person	22	21.8	
Stays with 2 to 4 other persons	57	56.4	
Stays with 5 or more other persons	22	21.8	
Household income			
Equal to or less than RM 3000	32	31.7	
RM 3001–5000	44	43.6	
More than RM 5000	25	24.8	
Formal education			
Primary education	20	19.8	
Secondary education	61	60.4	
Postsecondary or tertiary education	20	19.8	
Employment status			
Unemployed^a^	71	70.3	
Employed^b^	30	29.7	
Age at diagnosis (years)			49.7 (8.45)
Less than 50	46	45.5	
50 and older	55	54.5	
Duration of survivorship (years)			8.2 (5.72)
Up to 5	37	36.6	
More than 5	64	63.4	
Cancer stage at time of diagnosis			
Early stage (Stages I and II)	79	80.6	
Later stage (Stages III and IV)	19	19.4	
Do not know	3	3.0	
Current treatment status			
No current active treatment	73	72.3	
Undergoing active treatment	28	27.7	

^a^Unemployed: housewife/home maker, retiree.

^b^Employed: government/private employee, self-employed.

**Table 2:** Domain mean score (*N* = 101).

Rank	Domain	Mean (SD)	95% confidence interval for mean
1	Health systems and information	2.48 (0.800)	2.32–2.64
2	Psychological	2.01 (0.534)	1.91–2.12
3	Patient care and support	1.93 (0.509)	1.83–2.03
4	Physical and daily living	1.93 (0.623)	1.80–2.05
5	Sexuality	1.57 (0.651)	1.44–1.70

**Table 3:** Top ten items with highest “moderate to high” unmet needs (*N* = 101).

Rank	Item	% of respondents reporting needs	Domain
1	Having one member of hospital staff with whom you can talk to about all aspects of your condition, treatment, and follow-up	34.7	Health systems and information

2	Being given explanations on those tests about which you would like to get explanations	29.7	Health systems and information

3	Having access to professional counseling (e.g., psychologist, social worker, counselor, and specialist nurse) if you, family, or friends need it	27.7	Health systems and information

4	Being adequately informed about the benefits and side effects of treatments before you choose to have them	24.8	Health systems and information

5	Being informed about your test results as soon as feasible	20.8	Health systems and information

6	Being informed about cancer which is under control or diminishing (i.e., remission)	20.8	Health systems and information

7	Being given information (written information, diagrams, and drawings) about aspects of managing your illness and side effects at home	18.8	Health systems and information

8	Fears about the cancer spreading	16.8	Psychological

9	Being informed about things you can do to help yourself to get well	14.9	Health systems and information

10	Being treated in a hospital or clinic that is physically pleasant as possible	14.9	Health systems and information

**Table 4:** Top ten items with lowest “moderate to high” unmet needs (*N* = 101).

Rank	Item	% of respondents reporting needs	Domain
1	Feeling down or depressed	1.0	Psychological
2	Feelings of sadness	1.0	Psychological
3	Changes in sexual feelings	1.0	Sexuality
4	Changes in your sexual relationships	1.0	Sexuality
5	More choices about which hospital you attend	1.0	Patient care and support
6	Learning to feel in control of your situation	2.0	Psychological
7	Keeping a positive outlook	3.0	Psychological
8	Hospital staff attending promptly to your physical needs	3.0	Patient care and support
9	Pain	4.0	Physical and daily living
10	Worry that the results of treatment are beyond your control	4.0	Psychological

**Table 5:** Supportive care needs domain mean scores by sociodemographic and medical characteristics (*N* = 101).

	Mean (95% CI)				
	Supportive care need domains				
	Physical and daily living	Psychological	Sexuality	Patient care and support	Health system and information
*Respondent age*					
Below 60 years	2.07 (1.89, 2.25)	2.16 (2.01, 2.30)	1.77 (1.58, 1.95)	2.02 (1.87, 2.18)	2.52 (2.29, 2.74)
60 years and older	1.75 (1.60, 1.89)	1.82 (1.69, 1.96)	1.31 (1.17, 1.45)	1.81 (1.69, 1.93)	2.44 (2.21, 2.67)
*p* value	0.009	0.002	0.000	0.038	0.643
*Ethnicity*					
Chinese	1.84 (1.71, 1.97)	1.96 (1.84, 2.07)	1.48 (1.33, 1.64)	1.87 (1.76, 1.99)	2.48 (2.29, 2.66)
Malays and Sarawak indigenous group	2.18 (1.89, 2.47)	2.16 (1.91, 2.41)	1.81 (1.58, 2.04)	2.10 (1.89, 2.31)	2.50 (2.18, 2.82)
*p* value	0.017	0.095	0.028	0.051	0.898
*Marital status*					
Married	1.96 (1.80, 2.11)	2.00 (1.89, 2.11)	1.69 (1.54, 1.84)	1.92 (1.82, 2.02)	2.50 (2.33, 2.67)
Never married/widowed/divorced/permanently separated	1.82 (1.68, 1.96)	2.05 (1.76, 2.34)	1.11 (0.97, 1.25)	1.98 (1.65, 2.31)	2.42 (1.98, 2.86)
*p* value	0.181	0.725	0.000	0.714	0.710
*Cohabitation status*					
Lives alone or with 1 other person	1.86 (1.63, 2.10)	1.92 (1.68, 2.17)	1.42 (1.17, 1.68)	1.92 (1.66, 2.17)	2.40 (1.96, 2.85)
Stays with 2 to 4 other persons	1.96 (1.78, 2.14)	2.03 (1.90, 2.16)	1.69 (1.51, 1.87)	1.90 (1.78, 2.02)	2.51 (2.32, 2.71)
Stays with 5 or more other persons	1.91 (1.66, 2.15)	2.05 (1.78, 2.32)	1.39 (1.13, 1.66)	2.04 (1.78, 2.29)	2.48 (2.13, 2.83)
*p* value	0.827	0.678	0.097	0.555	0.866
*Household income*					
Equal to or less than RM 3000	1.94 (1.73, 2.15)	1.99 (1.78, 2.21)	1.36 (1.18, 1.55)	1.96 (1.75, 2.17)	2.34 (2.07, 2.62)
RM 3001–5000	2.05 (1.87, 2.22)	2.01 (1.88, 2.14)	1.62 (1.46, 1.78)	1.91 (1.79, 2.03)	2.39 (2.19, 2.60)
More than RM 5000	1.70 (1.41, 2.00)	2.04 (1.77, 2.30)	1.73 (1.36, 2.11)	1.94 (1.69, 2.18)	2.82 (2.42, 3.21)
*p* value	0.089	0.957	0.080	0.938	0.050
