# Corrigendum to “Unmet Supportive Care Needs among Breast Cancer Survivors of Community-Based Support Group in Kuching, Sarawak”

**DOI:** 10.1155/2017/4935107

**Published:** 2017-03-08

**Authors:** Emmanuel Joseph Fong, Whye Lian Cheah

**Affiliations:** Department of Community Medicine & Public Health, Faculty of Medicine & Health Sciences, Universiti Malaysia Sarawak, 94300 Kota Saramahan, Sarawak, Malaysia

In the article titled “Unmet Supportive Care Needs among Breast Cancer Survivors of Community-Based Support Group in Kuching, Sarawak” [1], an incomplete version of Table 5 was incorrectly included. The complete version is shown in Table 5.

Accordingly, the sentence “Respondents with primary education level reported a significantly lower domain mean score compared to those with postsecondary or tertiary education level across the domains of physical and daily living (1.71 versus 2.19, *p* = 0.012), sexuality (1.33 versus 1.95, *p* = 0.007), patient care and support (1.76 versus 2.21, *p* = 0.014), and health system and information (2.37 versus 3.00, *p* = 0.035)” in the Results section should be changed to “Respondents with primary education level reported a significantly lower domain mean score compared to those with postsecondary or tertiary education level across the domains of psychological (1.71 versus 2.19, *p* = 0.012), sexuality (1.33 versus 1.95, *p* = 0.007), patient care and support (1.76 versus 2.21, *p* = 0.014), and health system and information (2.37 versus 3.00, *p* = 0.035).

## Figures and Tables

**Table 5:** Supportive care needs domain mean scores by sociodemographic and medical characteristics (*n* = 101).

	Mean (95% CI)				
	Supportive care need domains				
	Physical and daily living	Psychological	Sexuality	Patient care and support	Health system and information
Respondent age					
Below 60 years	2.07 (1.89, 2.25)	2.16 (2.01, 2.30)	1.77 (1.58, 1.95)	2.02 (1.87, 2.18)	2.52 (2.29, 2.74)
60 years and older	1.75 (1.60, 1.89)	1.82 (1.69, 1.96)	1.31 (1.17, 1.45)	1.81 (1.69, 1.93)	2.44 (2.21, 2.67)
*p* value	0.009	0.002	0.000	0.038	0.643
Ethnicity					
Chinese	1.84 (1.71, 1.97)	1.96 (1.84, 2.07)	1.48 (1.33, 1.64)	1.87 (1.76, 1.99)	2.48 (2.29, 2.66)
Malays and Sarawak indigenous group	2.18 (1.89, 2.47)	2.16 (1.91, 2.41)	1.81 (1.58, 2.04)	2.10 (1.89, 2.31)	2.50 (2.18, 2.82)
*p* value	0.017	0.095	0.028	0.051	0.898
Marital status					
Married	1.96 (1.80, 2.11)	2.00 (1.89, 2.11)	1.69 (1.54, 1.84)	1.92 (1.82, 2.02)	2.50 (2.33, 2.67)
Never married/widowed/divorced/permanently separated	1.82 (1.68, 1.96)	2.05 (1.76, 2.34)	1.11 (0.97, 1.25)	1.98 (1.65, 2.31)	2.42 (1.98, 2.86)
*p* value	0.181	0.725	0.000	0.714	0.710
Cohabitation status					
Lives alone or with 1 other person	1.86 (1.63, 2.10)	1.92 (1.68, 2.17)	1.42 (1.17, 1.68)	1.92 (1.66, 2.17)	2.40 (1.96, 2.85)
Stays with 2 to 4 other persons	1.96 (1.78, 2.14)	2.03 (1.90, 2.16)	1.69 (1.51, 1.87)	1.90 (1.78, 2.02)	2.51 (2.32, 2.71)
Stays with 5 or more other persons	1.91 (1.66, 2.15)	2.05 (1.78, 2.32)	1.39 (1.13, 1.66)	2.04 (1.78, 2.29)	2.48 (2.13, 2.83)
*p* value	0.827	0.678	0.097	0.555	0.866
Household income					
Equal or less than RM 3000	1.94 (1.73, 2.15)	1.99 (1.78, 2.21)	1.36 (1.18, 1.55)	1.96 (1.75, 2.17)	2.34 (2.07, 2.62)
RM 3001–5000	2.05 (1.87, 2.22)	2.01 (1.88, 2.14)	1.62 (1.46, 1.78)	1.91 (1.79, 2.03)	2.39 (2.19, 2.60)
More than RM 5000	1.70 (1.41, 2.00)	2.04 (1.77, 2.30)	1.73 (1.36, 2.11)	1.94 (1.69, 2.18)	2.82 (2.42, 3.21)
*p* value	0.089	0.957	0.080	0.938	0.050
Formal education					
Primary education	1.87 (1.60, 2.14)	1.71 (1.51, 1.91)	1.33 (1.11, 1.55)	1.76 (1.57, 1.95)	2.37 (2.06, 2.69)
Secondary education	1.94 (1.78, 2.09)	2.05 (1.94, 2.17)	1.52 (1.39, 1.65)	1.90 (1.79, 2.00)	2.35 (2.19, 2.51)
Postsecondary or tertiary education	1.95 (1.59, 2.31)	2.19 (1.85, 2.53)	1.95 (1.49, 2.41)	2.21 (1.86, 2.56)	3.00 (2.44, 3.55)
*p* value	0.901	0.010^c^	0.006^d^	0.013^e^	0.005^f^
Employment status					
Unemployed	1.99 (1.68, 2.29)	2.18 (1.93, 2.42)	1.78 (1.46, 2.09)	2.13 (1.89, 2.38)	2.71 (2.34, 3.09)
Employed	1.90 (1.78, 2.03)	1.94 (1.83, 2.05)	1.48 (1.35, 1.60)	1.85 (1.75, 1.94)	2.39 (2.22, 2.55)
*p* value	0.532	0.042	0.081	0.034	0.111
Age at diagnosis (years)					
Less than 50	1.87 (1.68, 2.07)	2.10 (1.92, 2.27)	1.67 (1.44, 1.91)	1.97 (1.79, 2.15)	2.53 (2.27, 2.78)
50 and older	1.97 (1.81, 2.13)	1.94 (1.81, 2.07)	1.48 (1.35, 1.61)	1.90 (1.79, 2.01)	2.44 (2.24, 2.65)
*p* value	0.438	0.145	0.151	0.459	0.606
Duration of survivorship (years)					
5 or less	2.25 (2.03, 2.46)	2.26 (2.12, 2.41)	1.77 (1.52, 2.01)	2.11 (1.95, 2.27)	2.64 (2.38, 2.91)
More than 5	1.74 (1.61, 1.87)	1.86 (1.73, 2.00)	1.45 (1.31, 1.60)	1.83 (1.71, 1.95)	2.39 (2.19, 2.59)
*p* value	0.000	0.000	0.019	0.008	0.124
Current treatment status					
No current active treatment	1.77 (1.65, 1.90)	1.90 (1.77, 2.02)	1.47 (1.34, 1.61)	1.84 (1.72, 1.95)	2.37 (2.19, 2.55)
Undergoing active treatment	2.33 (2.07, 2.58)	2.31 (2.16, 2.46)	1.81 (1.51, 2.11)	2.19 (2.00, 2.37)	2.78 (2.48, 3.09)
*p* value	0.000	0.000	0.020	0.002	0.019
Cancer stage at time of diagnosis (*n* = 98)^g^					
Early stage (Stages I & II)	1.94 (1.79, 2.10)	2.01 (1.89, 2.12)	1.59 (1.44, 1.74)	1.92 (1.81, 2.04)	2.48 (2.30, 2.66)
Later stage (Stages III & IV)	1.92 (1.75, 2.08)	1.94 (1.73, 2.16)	1.58 (1.31, 1.85)	1.92 (1.67, 2.16)	2.42 (1.98, 2.85)
*p* value	0.860	0.627	0.964	0.949	0.752

*Bonferroni Post-Hoc Test*. ^c^Primary vs secondary education, *p* = 0.035; Primary vs post-secondary or tertiary education, *p* = 0.012, ^d^Primary vs post-secondary or tertiary education, *p* = 0.007; Secondary vs post-secondary or tertiary education, *p* = 0.026, ^e^Primary vs post-secondary or tertiary education, *p* = 0.014; Secondary vs post-secondary or tertiary education, *p* = 0.047, ^f^Primary vs post-secondary or tertiary education, *p* = 0.035; Secondary vs post-secondary or tertiary education, *p* = 0.004, ^g^Number of respondents included, *n* = 98, as 3 respondents could not recall their cancer stage at time of diagnosis. Domain mean score adjusted according to *n* = 98.
